# Energy Starvation Induces a Cell Cycle Arrest in *Escherichia coli* by Triggering Degradation of the DnaA Initiator Protein

**DOI:** 10.3389/fmolb.2021.629953

**Published:** 2021-05-13

**Authors:** Godefroid Charbon, Belén Mendoza-Chamizo, Christopher Campion, Xiaobo Li, Peter Ruhdal Jensen, Jakob Frimodt-Møller, Anders Løbner-Olesen

**Affiliations:** ^1^ Department of Biology, University of Copenhagen, Copenhagen, Denmark; ^2^ National Food Institute, Microbial Biotechnology and Biorefining, Technical University of Denmark, Kongens Lyngby, Denmark

**Keywords:** *Escherichia coli*, chromosome replication, initiation, DnaA protein, degradation, energy starvation

## Abstract

During steady-state *Escherichia coli* growth, the amount and activity of the initiator protein, DnaA, controls chromosome replication tightly so that initiation only takes place once per origin in each cell cycle, regardless of growth conditions. However, little is known about the mechanisms involved during transitions from one environmental condition to another or during starvation stress. ATP depletion is one of the consequences of long-term carbon starvation. Here we show that DnaA is degraded in ATP-depleted cells. A chromosome replication initiation block is apparent in such cells as no new rounds of DNA replication are initiated while replication events that have already started proceed to completion.

## Introduction

In *Escherichia coli*, like most other bacteria, chromosome duplication commences only once per cell cycle and at a defined cellular mass. Most regulatory inputs affect the function of the unique origin of replication *oriC* and/or the initiator protein DnaA (Leonard and Grimwade, 2015; Riber et al., 2016; Katayama et al., 2017; Hansen and Atlung, 2018). The origin of replication is composed of a duplex-unwinding region (DUE) and DnaA-oligomerization region (DOR). The DOR harbors recognition sites for DnaA and other regulatory proteins affecting DnaA–*oriC* structure and function. These include binding sites for nucleoid-associated proteins IHF and Fis, and the *oriC* methylation/sequestration proteins Dam/SeqA. The initiator protein DnaA is a multi-domain protein containing a C-terminal HTH DNA-binding site (Domain IV), an AAA+ ATPase domain responsible for DnaA multimerization on *oriC*, binding of single-stranded DNA-unwinding element and helicase loading on *oriC* (Domain III), and a domain responsible for interactions with helicase and other proteins and for DnaA dimerization (Domain I) (Zawilak-Pawlik et al., 2017). Domain III and Domain I are separated by a flexible linker with no apparent regulatory function (Domain II).

Changes in the abundance and activity of DnaA molecules are pivotal to achieve precise cell cycle–coupled initiations. During steady-state growth, the DnaA protein has a half-life of more than 24 h (Atlung and Hansen, 1999; Torheim et al., 2000); thus, its cellular concentration is essentially controlled at the synthesis level. This regulation allows for the buildup of the initiator protein pre-initiation, while transcriptional repression post-initiation arrests accumulation (Campbell and Kleckner, 1990; Theisen et al., 1993). Following replication, the hemi-methylated GATC sites located in the promoter region of *dnaA* are bound by the sequestration protein SeqA (Campbell and Kleckner, 1990; Lu et al., 1994) to inhibit *dnaA* transcription. Because *dnaA* is only located ∼40 kilo bases away from *oriC*, the repression is exerted shortly after initiation of replication. Approximately a fifth of a mass doubling time later, GATC sites are fully methylated by the Dam methyl transferase, and the repression is relieved. Additionally, DnaA represses its own expression (Atlung et al., 1985; Braun et al., 1985; Speck et al., 1999). Furthermore, the pool of DnaA proteins available for initiation is limited by binding sites spread throughout the chromosome which titrates DnaA away from *oriC* until enough molecules are present to occupy all of them (Hansen et al., 1991).

DnaA activity is set by its AAA+ ATPase (Domain III). In *E. coli*, DnaA is an intrinsically poor ATPase that is stimulated by regulatory elements (Katayama et al., 1998; Kato and Katayama, 2001; Fujimitsu et al., 2009; Kasho and Katayama, 2013). Like other members of AAA+ proteins, the binding of ATP and ADP specifies the conformation and multimerization status of the protein. DnaA forms a protein-DNA filament on *oriC* when it is bound to ATP (a requisite for unwinding of DUE and loading of the helicase). When bound to ADP, DnaA remains mainly monomeric and unable to nucleate from high-affinity binding sites to low-affinity binding sites located in the origin of replication. The initiator concentration and activity fluctuates during the cell cycle: the ratio of active to inactive (DnaA^ATP^/DnaA^ADP^) and protein concentration increases pre-initiation and decreases post-initiation (Kurokawa et al., 1999).

Several mechanisms govern the cellular DnaA^ATP^/DnaA^ADP^ ratio. Although DnaA has equal and extremely high affinity for ATP and ADP (Sekimizu et al., 1987), Apo-DnaA is expected to bind preferentially to ATP in the cell because ATP is about six to seven times more abundant than ADP. This means that Apo-DnaA molecules originating form *de novo* synthesis or “recycling” of DnaA^ADP^ will become mainly ATP bound. Post-initiation, the normally slow ATPase activity of DnaA is stimulated by the regulatory inactivation of DnaA (RIDA) complex composed of the Hda protein interacting with DNA-loaded *β*-clamp (Kato and Katayama, 2001). Thus, RIDA ensures that DnaA is converted to the inactive ADP-bound form. Additionally, three DNA-binding regions acting as chaperones promote the formation of specific DNA–DnaA structures that stimulate either DnaA ATPase activity (*datA* site) (Kasho and Katayama, 2013) or the release of ADP for formation of Apo-DnaA (*DARS1* and *DARS2* sites) (Fujimitsu et al., 2009). IHF and FIS also participate in these processes. The formation of Apo-DnaA through DARS, also called rejuvenation, permits the recycling of DnaA^ADP^ into DnaA^ATP^ because Apo proteins formed immediately bind ATP. Jointly, all these processes ensure 1) that DnaA molecules accumulate predominantly in an active form pre-initiation, 2) that DnaA accumulation ceases after initiation, and 3) that already synthesized molecules are inactivated and/or titrated away from the origin of replication after initiation. Concomitantly, DiaA, IHF, FIS, and SeqA either promote or prevent the formation of a DnaA–*oriC* complex capable of loading the helicase.

While the complex regulation of DNA replication has been extensively studied under steady-state growth conditions, much less has been reported on regulation during environmental changes or stress conditions. Most organisms possess systems that monitor DNA integrity and nutrient availability to prevent the start of chromosome replication under adverse conditions. In mammalian or yeast cells, checkpoints known as Restriction and Start, respectively, prevent the entry into the S phase if the nutrient requirements are not met (Johnson and Skotheim, 2013; Solaki and Ewald, 2018).

Since the binding of ATP in preference to ADP is essential for the activation of DnaA, we tested the impact of the energy status of the cell on DnaA function, that is, whether a low ATP/ADP ratio in the cell prevents the start of chromosome replication. By starving cells for carbon source or expressing an ATPase depleting ATP, we lowered the cellular ATP/ADP ratio. We observed a DNA replication block at the level of initiation following ATP starvation. Replication forks were not arrested but new initiations were. This results in the so-called replication run outs, with all replication rounds already commenced proceeding to completion. In time, cells end up containing only fully replicated chromosomes that are assumed more resilient to damages than those ongoing replication. The replication run out phenomenon can also be provoked by a general cessation of protein synthesis, triggered either by accumulation of alarmone (p)ppGpp or by treatment with antibiotics inhibiting ribosome or RNA polymerase function (Lark, 1972; Chiaramello and Zyskind, 1990; Schreiber et al., 1995). In these situations, the concentration of DnaA molecules fails to reach the minimal threshold necessary to trigger initiation. This can be further promoted by alteration of *oriC* topology when general transcription is affected (Kraemer et al., 2019; Riber and Lobner-Olesen, 2020). Here we show that stress caused by ATP starvation triggers degradation of DnaA, in contrast to protein synthesis arrest induced by chloramphenicol or rifampicin during which DnaA is stable over a period of 24 h.

## Results

### Degradation of DnaA in ATP-Starved Cells

**FIGURE 1:**
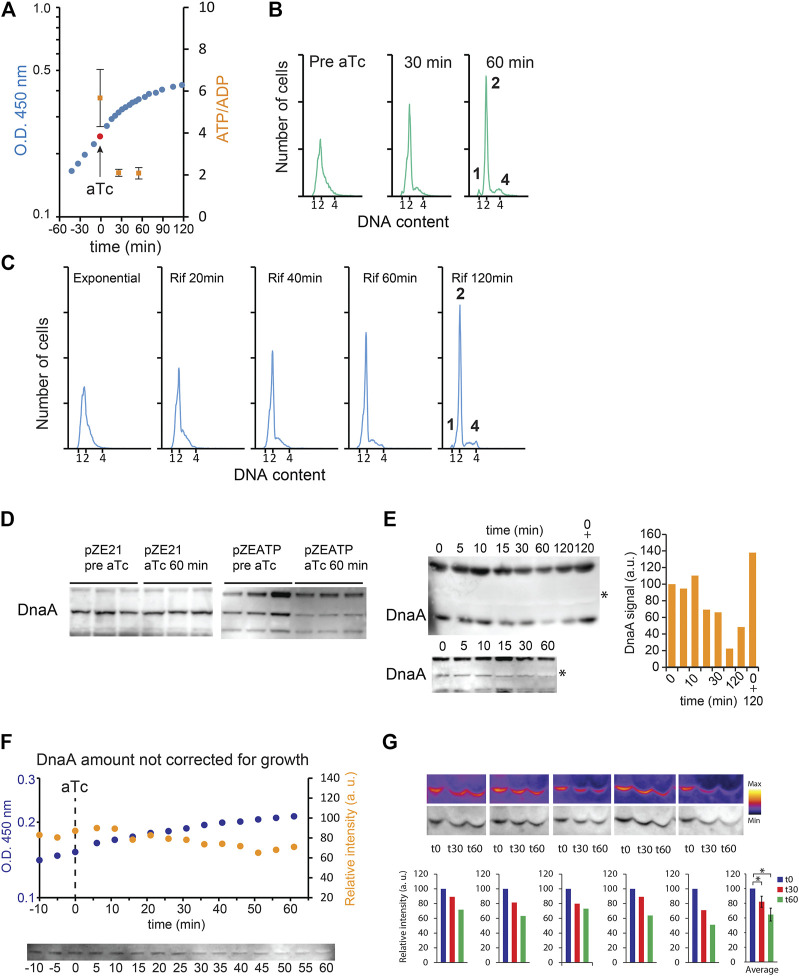
DnaA stability during ATP starvation. *E. coli* grown at 37°C in minimal medium supplemented with 0.2% glucose. **(A)** Growth curve of MG1655 Z1/pZATP. Induction of ATPase expression with 0.2 μg/ml anhydrotetracycline (aTc) is indicated by an arrow and a red ball. Time points where ATP/ADP was measured are shown in orange squares (SD +/− (n = 3)). **(B)** Flow cytometry analysis showing the DNA content of MG1655 Z1/pZATP cells taken prior to and following addition of aTc. Histogram displaying cells 60 min after ATPase induction shows complete replication run out with 1, 2, or 4 fully replicated chromosomes. **(C)** Flow cytometry analysis of cells treated with 300 μg/ml rifampicin and 36 μg/ml cephalexin. **(D)** DnaA protein level prior to and following addition of aTc in MG1655 Z1/pZE21 (control plasmid) and MG1655 Z1/pZATP. Samples were taken prior to and 60 min after addition of aTc. Protein samples were corrected for total protein concentration prior to electrophoresis. **(E)** The DnaA protein level prior to and following addition of aTc in MG1655 Z1/pZATP. The time point at which the samples were taken is indicated. Protein samples were corrected for total protein concentration prior to electrophoresis (top, long electrophoretic separation). 0 + 120 indicates that t_0_ and t_120_, samples are loaded together. * indicates unknown protein co-migrating with DnaA (bottom, shorter electrophoretic separation; right, quantification of the raw DnaA signal in the upper Western blot). **(F)** The DnaA protein level prior to and following addition of aTc in MG1655 Z1/pZATP without correction for total protein content. 100 μl of culture was harvested at each time points. The samples were centrifuged, and the whole cell pellet was loaded on the gel (top, optical density measurement of the growing culture (blue) and DnaA signal intensity from Western blot analysis (orange) are plotted on the graph; bottom, corresponding Western blot). **(G)** The DnaA protein level prior to and following addition of aTc in MG1655 Z1/pZATP without correction for the total protein content. One ml was harvested prior to and 30 min and 60 min after addition of aTc (five independently growing cultures). The samples were centrifuged, and the whole cell pellet was loaded on the gel. Histograms show DnaA signal intensity below the corresponding Western blot and the average of the measurements ± SD (n = 5),* = *p*< 0.05.

To address the role of cellular ATP/ADP ratio in determining the initiation of replication, we engineered cells that overproduce the catalytic F_1_ ATP synthase of *E. coli* (Koebmann et al., 2002). When uncoupled from the F_0_ part of the ATP synthase, the F_1_ ATP synthase acts as a potent ATPase that hydrolyzes ATP in the cell. Upon induction of the ATPase expression, the ATP/ADP ratio decreased from 5.7 (SD ± 1.4) to ∼2.0 (Figure 1A). Concomitantly, growth progressively slowed down, and initiation of DNA replication stopped shortly after ATPase induction: complete replication run out was seen already 1 h after ATPase induction (Figure 1B), that is, as fast as rifampicin-induced run out (Skarstad et al., 1986) (Figure 1C). Western blot analysis revealed that DnaA amounts decreased after induction of ATPase (Figure 1D), indicative of degradation of the initiator protein. We observed the presence of a protein migrating directly above DnaA in ATP-depleted extracts whose signal is quenched on some Western blots (Figure 1E). The co-migrating protein could affect the migration pattern of DnaA and its detection during Western blot analysis. Therefore, we analyzed samples after longer electrophoretic separation, and as a control, we mixed a sample pre-induction of ATPase with a sample post-induction. DnaA amounts were reduced after induction of ATPase even when the co-migrating protein was well separated from DnaA (Figure 1E). Furthermore, detection of DnaA by our anti-DnaA antibodies was not affected when an un-induced sample was mixed with an ATP-depleted sample (Figure 1E).

To rule out that the observed reduction in DnaA amount resulted from a cessation in DnaA transcription and continued growth during starvation, that is, dilution, we harvested fixed volumes of culture following ATP starvation and loaded whole samples on a polyacrylamide gel, that is, avoiding growth normalization. We observed a ∼40% reduction in DnaA content despite continued mass growth (Figures 1F,G), showing that degradation is at least in part responsible for reducing the amount of initiator protein.

**FIGURE 2:**
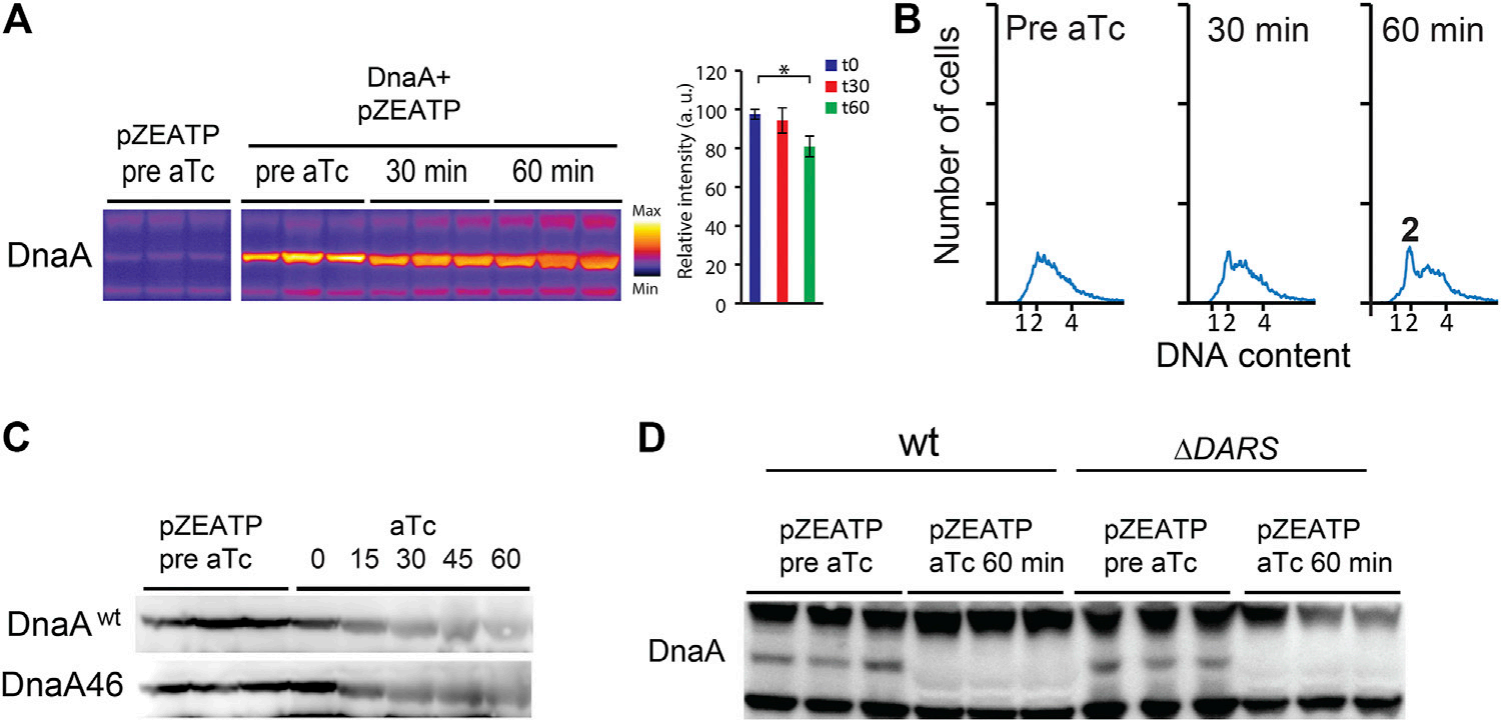
DnaA stability is not significantly affected by nucleotide binding. **(A)** The DnaA protein level prior to and following addition of aTc in MG1655 Z1/pZATP and MG1655 Z1/pZATP + pFH871 to overproduce DnaA (DnaA+). Cells were grown under the same conditions as in Figure 1. Protein samples were corrected for total protein concentration. The histogram shows DnaA signal intensity ± SD (n = 3),* = *p*< 0.05. **(B)** Flow cytometry analysis showing the DNA content of MG1655 Z1/pZATP + pFH871 (DnaA^+^) cells taken prior to and following addition of aTc. **(C)** The DnaA protein level prior to and following addition of aTc in MG1655 Z1/pZATP and MG1655 Z1 *dnaA46*/pZATP grown at 32°C in minimal medium supplemented with 0.2% glucose. Protein samples were corrected for total protein concentration. **(D)** The DnaA protein level prior to and following addition of aTc in MG1655 Z1/pZATP and MG1655 Z1 Δ*DARS1* Δ*DARS2*/pZATP grown in lysogeny broth (LB). Note that less proteins were loaded in the last three lanes as seen by weaker upper and lower cross-reacting bands.

Overproduction of DnaA (Figure 2A) overcame the replication block imposed by ATPase induction, visualized by partial and/or delayed replication run out after ATPase induction (Figure 2B). This indicates that it is the decrease in the amount of DnaA that results in an arrest in replication initiation in the wild type.

When the ATPase expression was induced in Δ*DARS1* Δ*DARS2* (lower DnaA^ATP^/DnaA^ADP^ ratio (Fujimitsu et al., 2009) or in *dnaA46* mutant cells, DnaA was likewise degraded (Figures 2C,D)). Since the DnaA46 mutant protein does not bind to ATP or ADP, these results could indicate that DnaA degradation does not depend on nucleotide binding.

### Degradation of DnaA is not Dependent on Lon and ClpP

**FIGURE 3:**
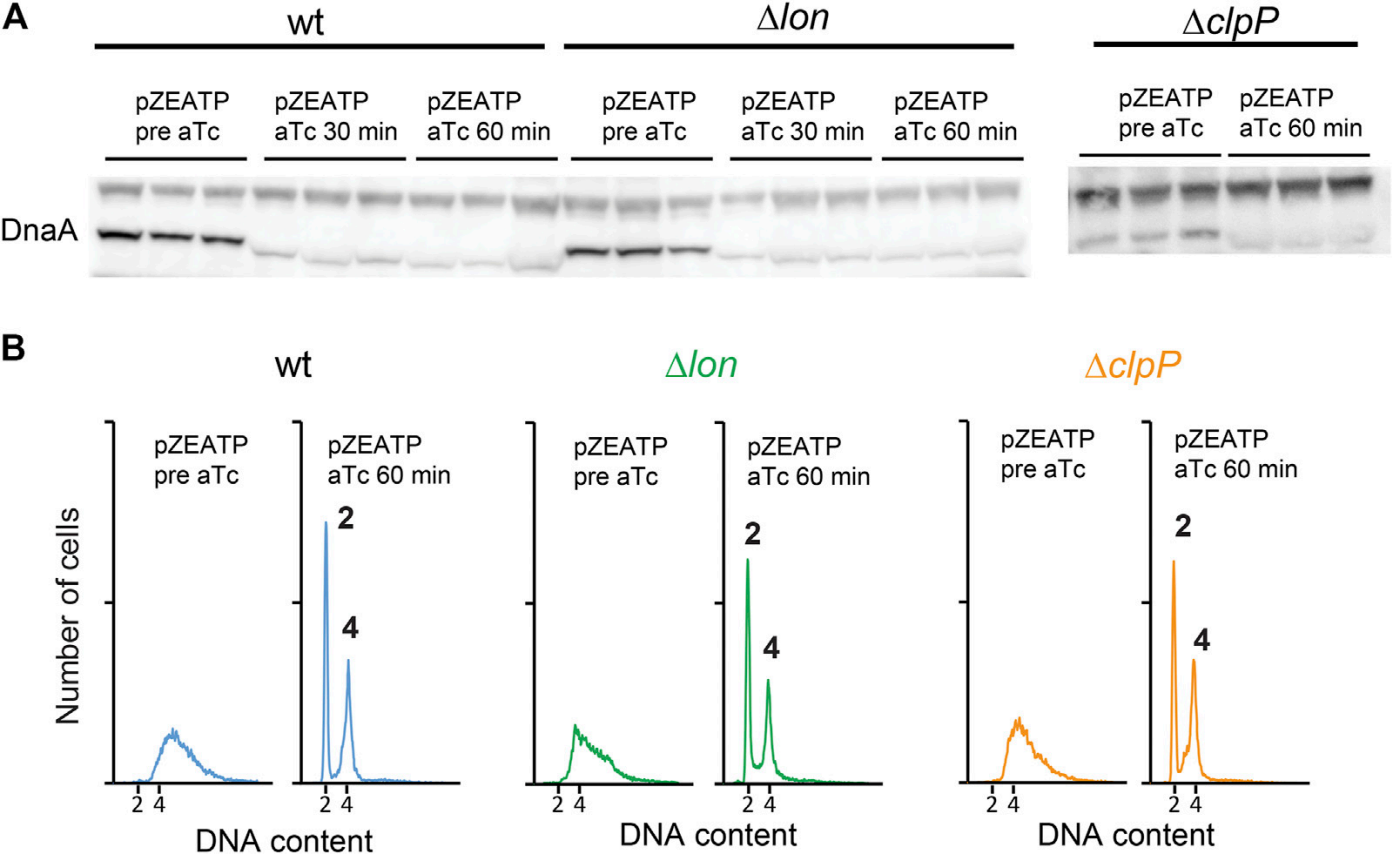
DnaA stability is not affected by ATP-dependent proteases Lon and ClpP. **(A)** The DnaA protein level prior to and following induction of ATPase in wild type, Δ*lon*, and Δ*clpP* grown at 37°C in LB. **(B)** Flow cytometry analysis showing the DNA content of wild type, Δ*lon*, and Δ*clpP* prior to and following addition of aTc to induce ATPase expression. Histogram displaying cells 60 min after ATPase induction shows complete replication run out with 2 or 4 fully replicated chromosomes.

Degradation of DnaA was previously described for *Caulobacter crescentus*. In this bacterium, DnaA (CcDnaA) has a much shorter half-life of approximately 40 min than *E. coli*, which is > 24 h . The CcDnaA stability is predominantly controlled by the ATP-dependent protease Lon that continuously degrades DnaA. Upon carbon starvation, the level of CcDnaA drops due to impairment in translation of *dnaA* mRNA, while the protease activity remains unchanged (Leslie et al., 2015). However, during proteotoxic stress, CcDnaA degradation is accelerated due to an increase in Lon activity under these conditions (Jonas et al., 2013). We induced expression of the ATPase in a *lon* mutant and observed that DnaA amounts are reduced (Figure 3A). Replication run outs were also observed (Figure 3B). Because the ClpAP protease also degrades DnaA in *C. crescentus* (Liu et al., 2016), we proceeded to induce the ATPase in a *clpP* mutant. Also in this case, DnaA concentration was reduced and initiation of replication arrested (Figures 3A,B). We further tested the stability of DnaA in a *hslV* mutant, and a triple *lon-hslV-clpP* mutant and found likewise that DnaA is not stabilized to the wild-type level, showing that none of the three proteases play a major role in controlling DnaA amounts in ATP-starved cells (Supplementary Figure S1).

### DnaA is Degraded in Carbon-Starved Cells

**FIGURE 4:**
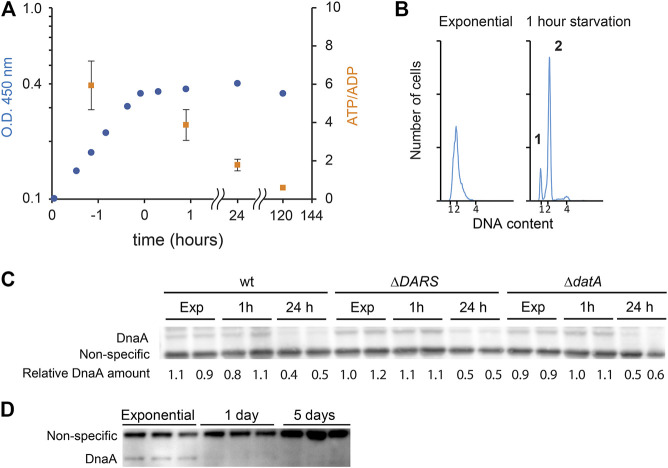
DnaA stability during carbon starvation. **(A)** Growth curve of MG1655 grown at 37°C in minimal medium supplemented with 0.02% glucose. Cells reached a plateau at O.D. 450 nm = ∼0.4. Time points where ATP/ADP was measured are shown as orange squares (SD +/− (n = 3)). **(B)** Flow cytometry analysis showing the DNA content of cells taken during exponential growth (third time point in panel **4A**), 1 h after glucose depletion (eighth time point in panel **4A**) showing cells with 1, 2, or 4 fully replicated chromosomes. **(C)** The DnaA protein level during exponential growth at 37°C in AB medium supplemented with 0.02% glucose and after 1 h or 1 day of carbon starvation in wild type, MG1655 Δ*DARS1* Δ*DARS2*, and MG1655 *datA* strains. **(D)** The DnaA protein level during exponential phase, 1 day, and 5 days after carbon starvation.

While cells maintain a relatively constant cellular energy state during normal growth, the ATP/ADP ratio is reduced during certain stress such as long-term stationary phase or carbon starvation (Chapman et al., 1971; Jensen et al., 1995). In order to starve *E. coli* for carbon source, cells were grown in minimal medium supplemented with a limited amount of glucose (Figure 4A). The ATP/ADP ratio declined from 5.9 (SD ± 1.3) during steady-state growth to 3.9 (SD ± 0.9) 1 h after glucose exhaustion from the growth medium. As expected (Chapman et al., 1971; Jensen et al., 1995), the ATP/ADP ratio in these carbon-starved cells declined further to 1.8 (SD ± 0.3) and 0.6 (SD ± 0.1) one day and five days after growth arrest, respectively. As previously reported for cells entering the stationary phase, initiation of DNA replication ceased concomitantly with growth arrest (Figure 4B) (Boye and Lobner-Olesen, 1991). This is seen by the appearance of fully replicated chromosomes 1 h after growth arrest: approximately the time it takes to duplicate chromosome in cells that had initiated replication immediately prior to growth arrest and similar to what is observed for rifampicin-induced run out (Figure 1C). Western blot analysis indicates that the DnaA protein level was unaffected for the first hour of carbon starvation, which is consistent with the fact that DnaA concentration remains constant in a wide range of steady-state growth conditions (Hansen et al., 1991) (Figure 4C). However, after 24 h of starvation, the DnaA level was decreased to 46% (SD ± 3.8) (Figures 4C,D), and since the half-life of DnaA far exceeds 24 h, this implies that DnaA is degraded during starvation periods. In comparison, the level of DnaA proteins after 24 h of treatment with the protein synthesis inhibitor chloramphenicol is unaffected (Atlung and Hansen, 1999; Torheim et al., 2000).

Since the ATP/ADP ratio in the cells is declining during starvation, we investigated the stability of DnaA in cells mutated in pathways controlling the DnaA^ATP^/DnaA^ADP^ ratio. Neither Δ*DARS1* Δ*DARS2* (lower DnaA^ATP^/DnaA^ADP^ ratio (Fujimitsu et al., 2009)) nor Δ*datA*-starved cells (higher DnaA^ATP^/DnaA^ADP^ ratio (Kasho and Katayama, 2013)) displayed a noticeable improvement in DnaA stability after long-term carbon starvation (∼50% decrease after 24 h, Figure 4C). This indicates that the binding of ATP or ADP would not play a significant role in DnaA protein stability in accordance with the findings in an *in vitro* study (Slominska et al., 2003).

## Discussion

In most organisms, nutrient availability is monitored by checkpoints that delay initiation of replication if cells are starved (Johnson and Skotheim, 2013; Solaki and Ewald, 2018). In *E. coli*, it is well known that stresses that block protein synthesis and growth specifically prevent initiation of chromosome replication. Consequently, a replication run out is observed in cells treated with chloramphenicol or rifampicin and when (p)ppGpp is induced. This results from a failure to accumulate the initiator protein DnaA to a level required to trigger initiation as a consequence of growth arrest (Riber and Lobner-Olesen, 2020).

DnaA is normally extremely stable with a half-life exceeding 24 h during steady-state growth; thus, most control is achieved by cell cycle fluctuation in *dnaA* transcription and DnaA protein activity. DnaA is active when bound to ATP, and it has long been assumed that newly synthetized protein will mainly bind to the most abundant nucleotide in the cell, that is, ATP. Although normally invariant, the ATP/ADP ratio decreased in cells experiencing prolonged carbon starvation with potential consequence for the DnaA^ATP^/DnaA^ADP^ level.

### An Energy-Dependent Checkpoint of DnaA Replication?

By specifically depleting ATP in the cells, we show that initiation is immediately blocked while already commenced replication rounds proceed to completion. The replication fork movement appears largely unaffected by ATP starvation, judging from these cells’ ability to make replication run out at a speed similar to rifampicin-treated cells (Skarstad et al., 1986) where the ATP level is actually elevated (Link et al., 2015). The initiation block in ATP-starved cells resulted from reduced DnaA amounts as it was not observed in cells overproducing DnaA. While we show that DnaA is indeed degraded in ATP-starved cells (Figures 1F,G), we cannot rule out a contribution from dilution as well. The latter would result from a cessation of DnaA synthesis while growth continued. Nevertheless, a reduction in DnaA could serve to prevent the next round of replication until conditions are favorable and DnaA is resynthesized. This would mechanistically resemble a checkpoint, and we speculate that cells experiencing transient energy starvation could be subject to this regulation.

During ATP starvation, the nucleotide-binding status of DnaA does not appear to play a significant role. The amount of Apo-DnaA proteins mimicked by DnaA46 is reduced during the first 15 min of ATP starvation as also observed in wild-type cells. Unlike what is seen in *C. crescentus*, the degradation in *E. coli* is not performed by the ATP-dependent proteases Lon, ClpP, or HslV. The protease(s) and the mechanism of degradation of the initiator protein are unknown and are subject to further investigation, but the overall picture suggests that DnaA is degraded upon ATP depletion.

We speculate that the apparent slow rate of DnaA depletion during ATP starvation results in part from the inability of ATP-dependent proteases to degrade DnaA efficiently and in part from continued *de novo* DnaA synthesis in the starved cells.

### DnaA Degradation Triggered by Long-Term Carbon Starvation

We show here that upon carbon starvation, initiation is promptly arrested. Since the ATP/ADP ratio is nearly unaffected during the first hour of starvation, this initiation block is not related to the change in the energy level but may be explained by an arrest in initiator protein synthesis and failure to accumulate the initiator protein to the level required for initiation. In other starvation conditions, the DnaA level has been reported to be constant or reduced due to (p)ppGpp transcriptional repression, depending on how fast growth is arrested after stress. Upon prolonged starvation, we observe that DnaA levels decreased. Because there is little or no growth, this can only be explained by degradation of the initiator. However, DnaA degradation takes several hours to occur, as does ATP depletion. In a population of steady-state growing cells, about 30 percent of the DnaA molecules are bound to ATP, while the remainder are bound to ADP (Kato and Katayama, 2001). The nucleotide binding could potentially influence protein stability. This does not appear to be the case during ATP starvation as mutations affecting the DnaA^ATP^/DnaA^ADP^ balance do not appear to influence DnaA stability. Since DnaA is capable of exchanging ATP and ADP through the DARS rejuvenation and DDAH process, the nucleotide-binding status of DnaA would mostly affect the speed at which DnaA is degraded immediately after starvation. Presumably, as 70 percent of DnaA molecules are bound to ADP prior to starvation, a fast decrease in the protein level could be achieved by targeting the inactive form of the initiator. We see no change in protein concentration during the first hour of starvation or in a DARS mutant in which DnaA^ATP^/DnaA^ADP^ balance is decreased. Together, this suggests that the nucleotide-binding status of DnaA may not play a predominant role in controlling its stability. This is in contrast with a recent report showing that Lon specifically degrades DnaA^ADP^ during starvation stress (Gross and Konieczny, 2020). *In vitro*, Lon specifically degrades DnaA^ADP^ in the presence of polyphosphate only (Gross and Konieczny, 2020). We do not observe stabilization of DnaA during ATP depletion in a *lon* mutant. This is consistent with the fact that Lon function is ATP dependent. We do not exclude that Lon together with other proteases participate in the degradation of DnaA, but its action is dispensable, consistent with reports showing that the stability of thermosensitive *dnaA* mutants is increased in the absence of Lon, ClpP, or HlsV (Slominska et al., 2003). Alternatively, because Lon only degrades DnaA^ADP^ in the presence of polyphosphate, it is possible that polyphosphate accumulation is negligible in the time frame of ATP depletion (15–30 min). We note, however, a difference in the experimental procedure as loading of protein samples is corrected per total protein (present study) instead of cell number (Gross and Konieczny, 2020). This could become relevant as cell mass is known to decrease upon entry in the stationary phase.

### A Tight Control in Periods of Need

The regulatory role of the energy-dependent degradation of DnaA is elusive. Since the initiation block is already triggered by a growth arrest, the need for an additional level of control appears intuitively redundant. We speculate that ATP starvation could trigger specific cleavage of ATP-consuming enzymes as a general strategy to conserve energy. Being an ATPase, DnaA would belong to this group of enzymes. Alternatively, DnaA stability could be maintained by the activity of ATP-consuming chaperones that would fail to achieve this role in ATP-starved cells. This could explain reports showing that GroEL and DnaK affect the initiation of replication or DnaA (Jenkins et al., 1986; Sakakibara, 1988). Last, the degradation of DnaA could ensure a tight and fast block of initiation of replication. The need for such level of regulation would be to avoid an otherwise deleterious replication event during prolonged stress conditions. In those situations, DNA replication would be accompanied by accumulation of DNA strand breaks lethal for the cells.

## Materials and Methods

### Medium

Cells were grown in lysogeny broth (LB) medium or AB minimal medium supplemented with 10 µg ml^−1^ thiamine and either 0.2% or 0.02% glucose (for carbon starvation experiments). Experiments were performed at 37°C unless otherwise indicated. For selection, the following concentrations of antibiotics were used: kanamycin, 50 μg/ml; chloramphenicol, 20 μg/ml; and ampicillin, 150 μg/ml.

### Bacterial Strains and Plasmids

All strains used are listed in Supplementary Table S1.

The Z1 locus encoding the TetR repressor and resistance to spectinomycin at attλ (Diederich et al., 1992) was moved by P1 transduction using a lysate of MG1655 Z1 (*F* ^*−*^, *lambda* ^*-*^, *rph-1*, *lacI* ^*q*^, *PN25-tet* ^*R*^, and *Sp* ^*R*^) (Bury-Mone et al., 2009).

The clpP mutation from strain JW0427 (Baba et al., 2006) was PCR-amplified using primers CTG​ATA​ATC​CGT​CCA​TAA​GG and GCG​TTG​TGC​CGC​CCT​GGA​TA. The amplicon was transformed in MG1655 strain bearing the pKD46 plasmid (Datsenko and Wanner, 2000).

The hslV mutation (JW3903) was moved by P1 transduction.

A MG1655 *dnaA46* mutant (ALO6830) strain lacking tetracycline resistance and in which *tnaA* gene was reintroduced was created using a P1 lysate of wild-type MG1655 and strain ALO2342 (MG1655 *dnaA46*, *tnaA:Tn10*). ALO6830 was selected for growth on minimal medium containing tryptophan, tetracycline sensitivity, and thermosensitivity.

Plasmid pFH871  (Atlung et al., 1985) is a pACYC184-derived plasmid carrying *dnaA* with its own promoter.

### Plasmid Construction

pZEATPAGD was constructed by amplification of *E. coli atpAGD* operon using primers GGG​GTA​CCA​TGC​AAC​TGA​ATT​CCA​CCG​AAA​T and GCG​GGA​TCC​CTC​CGA​TTA​AGG​CGT​TAA​AG. The amplicon and plasmid pZE21 were cut with restriction enzyme *Kpn*I and *BamH*I, and then ligated to create pZEATPAGD.

### ATP Measurement

Quantification of ATP and ADP was performed as previously described (Koebmann et al., 2002). 500 μl culture was mixed with pre-warmed 60°C phenol equilibrated with 10 mM Tris HCl, pH 8.0, and 1 mM EDTA (Sigma P4557l), incubated for 5 min at 60°C with intermittent whirl mixing, and then stored at −20°C. Then aqueous phase was extracted twice with phenol and chloroform. ATP/ADP ratios were measured using a luciferin–luciferase ATP Kit (A22066 Invitrogen™), pyruvate kinase (Sigma P0294), and phosphoenolpyruvate (Sigma 10108294001). The luminescence was measured using the Infinite 1M1000 PRO microplate reader (TECAN).

### Western Blot

10–20 ml samples were harvested, placed on ice for 5 min, and centrifuged at 4000 x g for 7 min at 4°C. The supernatant was discarded, and the cell pellet was immediately stored at −20°C. Samples were resuspended in cold 200 μl PBS containing protease inhibitor (cOmplete™, Mini, EDTA-free Protease Inhibitor Cocktail, #11836170001) and incubated for 5 min at 95°C. The samples were then lyzed by sonication at 4°C using a Bioruptor^®^ Plus sonication device. Protein concentration was estimated using the Bradford assay (Sigma B6916), and total protein amount was adjusted to the same level in all samples prior to SDS-PAGE electrophoresis. For analysis of samples not normalized for growth, 0.1 or 1 ml culture as indicated in figure legends was harvested, placed on ice for 5 min, and centrifuged at 10,000 x g for 5 min at 4°C. The supernatant was discarded, and the cell pellet was immediately stored at −20°C. The pellet was resuspended in 15 μl PBS and 15 μl loading buffer, and incubated for 5 min at 95°C. Electrophoresis was performed using Precast 4–12% Bis-Tris Gel NuPAGE™ or 20 cm handcast Tris-HCl 10% acrylamide gels. Polyclonal anti-DnaA antibodies were used to detect DnaA (Charbon et al., 2011). Western blot quantification and analysis was performed using ImageJ software.

### Flow Cytometry

500 µl culture was centrifuged for 5 min at 8,000 × *g* at 4°C, and the supernatant was discarded. The cells were resuspended in 100 μl of cold 10 mM Tris pH 7.5, then fixed by adding 1 ml of 77% ethanol, and stored at 4°C until use.

Prior to flow cytometry analysis, the samples were centrifuged at 15,000 × *g* for 15 min. The supernatant was discarded, and the pellet was resuspended in 150 µl DNA staining solution (90 μg/ml mithramycin, 20 μg/ml ethidium bromide, 10 mM MgCl2, and 10 mM Tris pH 7.5). Samples were incubated for a minimum of 10 min prior to analysis. Flow cytometry was performed using Apogee A10 Bryte.

## Data Availability

The raw data supporting the conclusions of this article will be made available by the authors, without undue reservation.
