# Supplementary material for: Energy Starvation Induces a Cell Cycle Arrest in Escherichia coli by Triggering Degradation of the DnaA Initiator Protein
Source: Front Mol Biosci. 2021 May 13;8:629953. doi: 10.3389/fmolb.2021.629953 (PMC8155583; doi:10.3389/fmolb.2021.629953)
Supplement: Supplementary file 1 [file Table1.DOCX]

**Table S1.**

| **Strain Name** | **Genotype** | **Reference/Source** |
| --- | --- | --- |
| ALO1825 | F^-^ λ^-^ *rph-1* | (1) |
| DA20176 | F^-^ λ^-^ *rph-1* *ilvG*- *rfb-50* Δ*sraA*-*lon* | (2) |
| ALO7532 | *clpP*::*kan ^a^* | This work |
| ALO4835 | Δ*DARS1* Δ*DARS2 ^a^* | This work |
| ALO2381 | *datA::kan ^a^* | This work |
| ALO6830 | *dnaA46 ^a^* | This work |
| ALO7464 | *Z1* ^a^ | This work |
| ALO7530 | Δ*sraA*-*lon Z1* ^b^ | This work |
| ALO7534 | Δ*clpP Z1* ^a^ | This work |
| ALO7473 | Δ*DARS1* Δ*DARS2 Z1* ^a^ | This work |
| ALO6830 | *dnaA46* *Z1* ^a^ | This work |
| ALO7698 | Δ*clpP* Δ*hslV* Δ*sraA*-*lon* *Z1* ^a^ | This work |
| ALO7679 | Δ*hslV* *Z1* ^a^ | This work |

^a^ Genotype otherwise as MG1655 (ALO1825)

^b^ Genotype otherwise as DA20176

1. Guyer MS, Reed RR, Steitz JA, Low KB. Identification of a sex-factor-affinity site in *E. coli* as gamma delta. Cold Spring HarbSympQuantBiol. 1981;45:135-40.

2. Nicoloff H, Andersson DI. Lon protease inactivation, or translocation of the lon gene, potentiate bacterial evolution to antibiotic resistance. Mol Microbiol. 2013;90(6):1233-48.
