# Supplementary material for: Energy Starvation Induces a Cell Cycle Arrest in Escherichia coli by Triggering Degradation of the DnaA Initiator Protein
Source: Front Mol Biosci. 2021 May 13;8:629953. doi: 10.3389/fmolb.2021.629953 (PMC8155583; doi:10.3389/fmolb.2021.629953)
Supplement: Supplementary file 2 [file DataSheet1.PDF]

Supplemental Figure 1

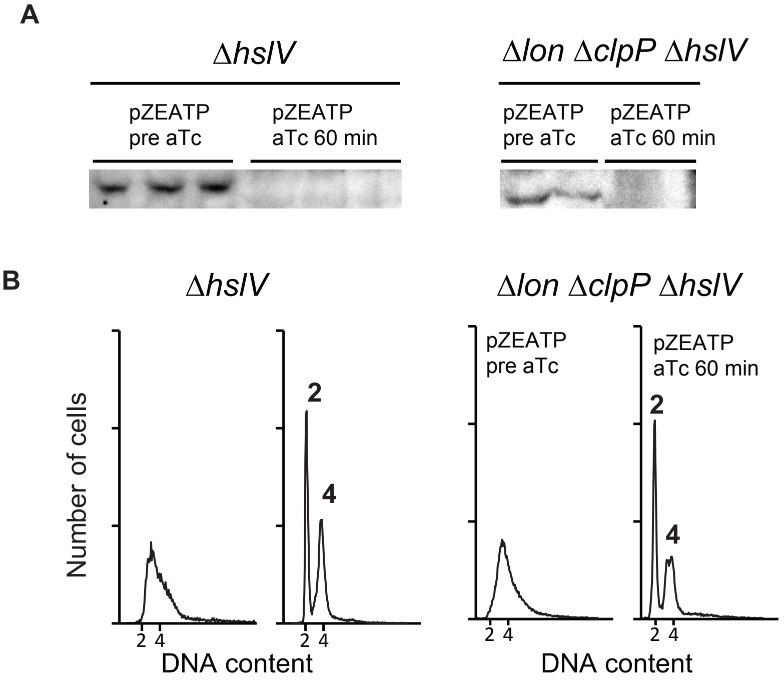

Supplemental Figure 1. DnaA stability is not affected in *hslV* or *lon-ClpP-hslV* mutant after ATPase induction.

A) DnaA protein level prior to and following induction of ATPase Δ*hslV* and Δ*lon-ClpP-hslV* grown at 37 °C in LB.

B) Flow cytometry analysis showing the DNA content of wild type, Δ*hslV* and Δ*lon-ClpP-hslV* prior to and following addition of aTc to induce ATPase expression. Histogram displaying cells 60 minutes after ATPase induction shows complete replication runout with 2 or 4 fully replicated chromosomes.
